# The Impact of Hyperuricemia on the Progression of Atherosclerotic Cardiovascular Disease

**DOI:** 10.7759/cureus.94371

**Published:** 2025-10-11

**Authors:** Marika Mdivnishvili, Maia Kharebashvili, Vakhtang Chumburidze, Kakha Nadaraia, Lali Gujejiani, Ketino Virkovi

**Affiliations:** 1 Medicine, Vakhtang Bochorishvili Clinic, Tbilisi, GEO; 2 Cardiotherapy, Open Heart-University Hospital, Tbilisi, GEO; 3 Clinical Research, Chapidze Heart Disease Center, Tbilisi, GEO; 4 Clinical and Interventional Cardiology and Therapy, Tbilisi State University (TSU) Clinic "Vivamedi", Tbilisi, GEO; 5 Cardiology, Open Heart-University Hospital, Tbilisi, GEO; 6 Cardiology, East European University, Tbilisi, GEO; 7 Medicine, Caucasus International University, Tbilisi, GEO

**Keywords:** asymptomatic hyperuricemia, atrial fibrilation, coronary artery disease, diabetes, metabolic syndrome

## Abstract

Hyperuricemia refers to elevated levels of serum uric acid (SUA) beyond the normal physiological range in men and women. It is caused by increased endogenous synthesis, decreased renal clearance, or a combination. While hyperuricemia has long been associated with gout, new evidence suggests that it is a significant risk factor in the pathophysiology of a variety of cardiovascular and metabolic disorders, including hypertension, metabolic syndrome, coronary artery disease, heart failure, type 2 diabetes mellitus, and chronic kidney disease. The increasing global frequency of hyperuricemia has sparked scientific and clinical interest in the possible mechanistic relationship between elevated SUA levels and cardiovascular morbidity. Hyperuricemia's pathological mechanisms, including oxidative stress, endothelial dysfunction, and systemic inflammation, may lead to vascular remodeling, atherosclerotic plaque progression, and increased cardiovascular mortality.

This case report emphasizes the need for increased clinical awareness of the cardiovascular dangers associated with asymptomatic hyperuricemia. Its purpose is to investigate the potential link between elevated SUA and the severity of coronary artery involvement, emphasizing the importance of early detection and treatment of hyperuricemia as a modifiable cardiovascular risk factor.

## Introduction

Hyperuricemia is known to be associated with cardiovascular disease (CVD), such as coronary artery disease (CAD), stroke, and hypertension. Still, the role of serum uric acid (SUA) as an independent risk factor for CVD remains unclear. Studies have demonstrated that hyperuricemia is not only a risk factor for gout but also associated with cardiovascular (CV) damage. According to data from the U.S. National Health and Nutrition Examination Survey (NHANES) [1], approximately 21% of adults, or 43 million individuals, are affected by hyperuricemia. A meta-analysis of prospective cohort studies found a 10% increase in mortality due to coronary artery disease [2]. Studies have also established an association between hyperuricemia and the development of arrhythmias [3].

Studies conducted in Japan [4] have demonstrated that elevated uric acid (UA) levels may contribute to the development of atrial fibrillation (AF), as well as increase the risk of ventricular arrhythmias.

Therefore, effective and timely intervention in the management of hyperuricemia is vital to prevent serious or life-threatening outcomes. The often asymptomatic presentation of hyperuricemia complicates early identification, which can lead to delays in treatment. Prolonged periods of untreated hyperuricemia significantly heighten the risk of CV complications, thereby emphasizing the necessity for early detection and prompt management. Elevated SUA levels have been increasingly recognized as an independent biomarker and potential contributor to CVD pathogenesis. Numerous epidemiological and clinical studies have established a strong association between hyperuricemia and various CV conditions, including hypertension, coronary artery disease (CAD), cerebrovascular disease, vascular dementia, and pre-eclampsia [5]. The prevalence of hyperuricemia is notably higher among individuals with coexisting conditions such as chronic kidney disease, post-menopausal status, and longstanding hypertension, all of which are themselves risk factors for adverse CV outcomes.

Recent research has further underscored the prognostic value of elevated UA levels in predicting CV morbidity and mortality. For instance, Zuo et al. demonstrated a significant correlation between hyperuricemia and increased risk of CAD and its related complications [2]. Despite the availability of effective pharmacologic interventions to reduce SUA levels, the clinical implications of treating asymptomatic hyperuricemia remain an area of ongoing investigation. Understanding the pathophysiological role of UA in CVD is essential for developing targeted strategies to mitigate its impact on vascular health.

Despite mounting evidence of this association, there is still no agreement on the use of urate-lowering treatments, such as xanthine oxidase inhibitors, in patients with asymptomatic hyperuricemia, due to a lack of randomized controlled trial data proving CV benefit. As a result, the illness remains undetected and untreated in clinical settings, particularly among high-risk populations. This case report highlights the importance of proactive screening and better monitoring of SUA levels in people with multiple CV risk factors, even without gout-related symptoms.

## Case presentation

This case involves a 57-year-old man with a significant medical history of poorly controlled hypertension (ranging from 160 to 180 mmHg) and type 2 diabetes mellitus, which was diagnosed five years ago and is currently managed with oral hypoglycemics (metformin, sitagliptin). On August 14, 2024, at 13:00, the patient arrived at the hospital complaining of substernal chest pain at rest that radiates to the left upper limb and is accompanied by shortness of breath, hypotension, generalized weakness, diaphoresis, and palpitations. Furthermore, over the previous two days, he noticed that regular physical exercise caused chest discomfort, indicating a likely increase in symptom severity and frequency.

He also described having a similar experience about a month prior, which he treated symptomatically without seeking professional help. According to the patient, these symptoms had become increasingly severe, necessitating immediate medical intervention.

Upon arrival at the emergency department, the patient underwent a preliminary evaluation. Following administration of nitroglycerin, the intensity of the retrosternal pain diminished, but the discomfort in the chest persisted. His vital signs included a heart rate of 128 bpm, blood pressure of 100/60 mmHg, respiratory rate of 22 breaths per minute, and oxygen saturation of 97% on room air. His body temperature was recorded at 36.6°C. Lung auscultation revealed vesicular breath sounds, and abdominal examination was unremarkable, with no hepatomegaly, palpable splenomegaly, or peripheral edema.

**Figure 1:**
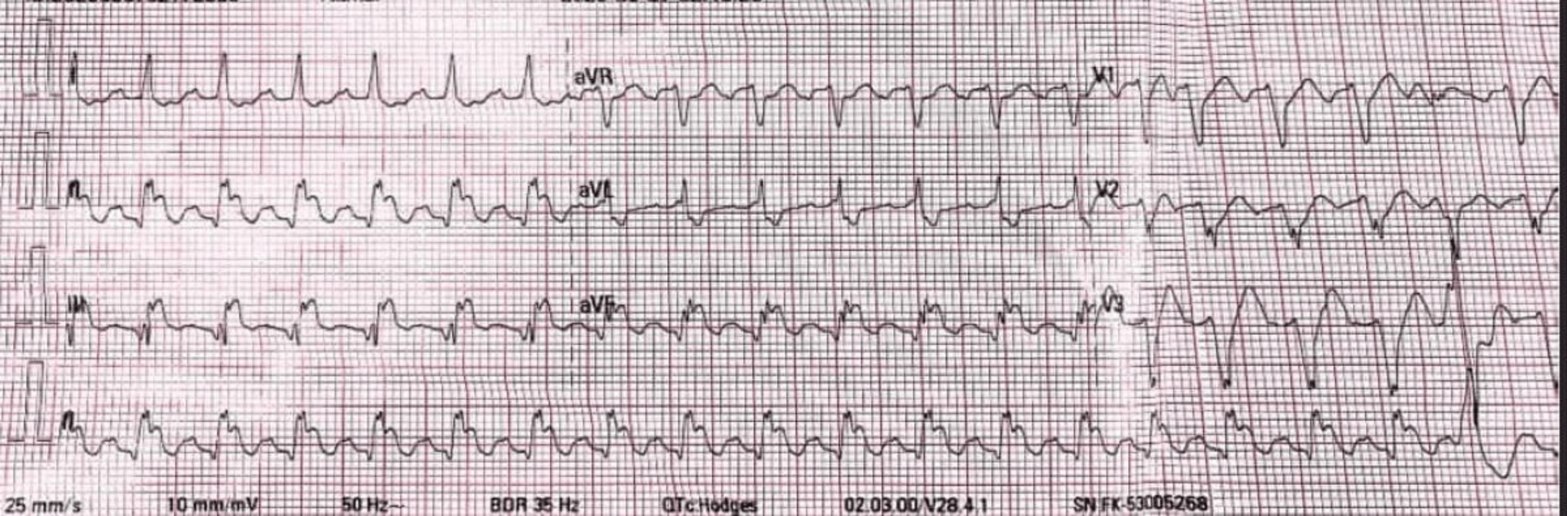
ECG showing atrial fibrillation, 2:1 left bundle branch block, ventricular extra systoles, and ST elevation in leads II, III, and aVF: T V4 V5 V6

**Figure 2:**
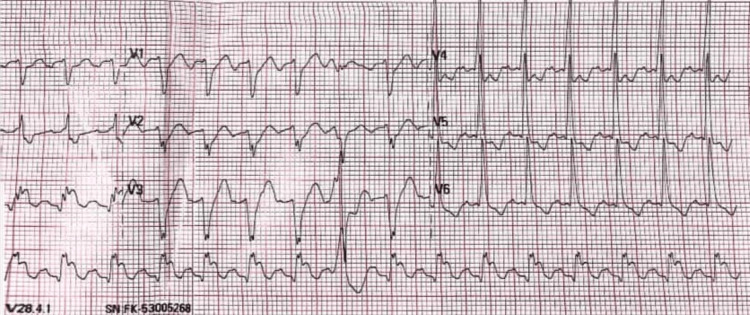
ECG showing atrial fibrillation, 2:1 left bundle branch block, ventricular extrasystoles, and ST elevation in leads II, III, and aVF: T V4 V5 V6

At 13:25, an electrocardiogram (ECG) revealed AF, ST elevation in leads II, III, and aVF, and a heart rate of 141 bpm (Figures 1, 2). Chemical cardioversion successfully restored sinus rhythm. The post-cardioversion ECG showed sinus rhythm with ventricular bigeminy and T-wave inversions in leads II, III, and aVF. The ejection fraction was measured at 40%.

Laboratory investigations included assessments of UA, creatinine, blood glucose, electrolytes, troponin, complete blood count (CBC), and lipid blood test. All results were within normal limits except positive troponin and elevated UA levels (Table 1). Specific treatment was initiated to lower UA levels, with allopurinol prescribed by current guidelines for hyperuricemia management (Table 2).

**Table 1:** Laboratory results

Investigation	Result	Normal Range
CRP	5.6	<6 mg/L
Uric Acid	951	200-420 µmol/L
Creatinine	103	62-123 µmol/L
BCT	8	4-9 minutes (assumed)
Activated Plasma Recalcification Time (APRT)	130	60-190 seconds
PT Index %	88 %	80%-100%
INR	1.15	1.0-1.2
Fibrinogen	Negative	Negative
Ethanol Sample	Negative	Negative
Blood Clot Retraction	Normal	Normal
Fibrinogen Concentration	2.9	2-4 g/L
Hemoglobin/HGB	142	140-160 g/L
Erythrocyte/RBC	5.3	4.0-5.0x10¹²/L
Leukocyte/WBC	8.2	4.0-9.0x10⁹/L
Thrombocyte/PLT	315	180-320x10⁹/L
NEUT band	2	1-6%
NEUT Segm	59	47-72%
Eosinophil	2	0.5-5%
Lymphocyte	31	19-37%
Monocyte	6	3-11%
ESR	7	2-10 mm/hr
HCT	45	40-48%
MCV	86	80-96fL
MCH	26.7	26-34pg
MVHV-Hb	312	300-500
RDW	14.7	12-14%
MPV	7.2	8-15fL
Troponin	0.053	<0.5 ng/mL

**Table 2:** Medical treatment

Medical treatment	
Rosuvastatin	20mg 1x p/o within three months.
Clopidogrel	75 mg 1x p/o within three months.
Metoprolol	25mg 2x p/o within three months.
Amiodarone	200mg 2x p/o within 10 days
Fluconazole	150mg 1x p/o
Comb. drug perindopril arginine, amlodipine	10mg/10mg 1x p/o with blood pressure control
Rivaroxaban	15mg 1x p/o within three months
Allopurinol	100mg 1x p/o within four weeks
Comb. drug sitagliptin phosphate Metformin Hydrochloride	50mg/1000mg 1x p/o

Based on clinical presentation and diagnostic findings, the patient was diagnosed with ischemic heart disease, specifically unstable angina. Despite anti-ischemic and anticoagulant therapy, the patient continued to experience angina. Given his high-risk profile and evidence of ischemia on the ECG, coronary angiography was performed on August 5, 2024, at midnight. The angiogram revealed multiple coronary artery lesions, and coronary artery bypass grafting (CABG) was subsequently performed. After a few hours, the patient's ECG showed recurrent changes, and the ECG showed typical atrial flutter. 

Postoperatively, the patient demonstrated progressive clinical improvement, stabilizing vital signs and normalizing relevant laboratory parameters. His recuperation was uneventful, and he responded well to cardiac and metabolic treatment. By the end of the week, the patient was discharged in stable condition, with recommendations for continued outpatient medical therapy and follow-up.

The patient displayed typical symptoms of unstable angina, such as left-sided chest discomfort, palpitations, and shortness of breath. ECG results showed ischemia alterations, while coronary angiography revealed several severe coronary artery lesions. As a result, CABG was undertaken. Given the severity and symptomatic nature of the obstructions, surgical intervention was preferable to medical treatment alone. The patient's condition improved symptomatically, and the test results showed improvement after surgery.

## Discussion

UA, the byproduct of purine metabolism catalyzed by the enzyme xanthine oxidase, is receiving increased interest for its potential role in the etiology of CV and cardiometabolic illnesses. Large-scale epidemiological studies have repeatedly linked elevated SUA levels to an increased risk of hypertension, type 2 diabetes, and coronary heart disease [6,7]. 

Growing evidence supports the use of blood UA not only as a biomarker for CVD, but also as a measure of cumulative CV risk burden. Although the precise mechanisms underlying this association are not fully understood, several pathophysiological pathways have been proposed. UA has increasingly been associated with CVD through various mechanisms, including oxidative stress, widespread inflammation, and changes in atrial structure. These factors lead to endothelial dysfunction by inhibiting nitric oxide production and damaging the kidney's microvasculature and vascular inflammation, leading to high blood pressure and speeding up atherosclerosis [8]. Elevated UA levels can trigger the NLRP3 inflammasome in vascular smooth muscle cells (VSMCs) via increased phagocytosis, releasing inflammatory cytokines like IL-1β and IL-18. Notably, IL-1β plays a crucial role as a mediator of the inflammatory process, further contributing to vascular inflammation and the development of atherosclerosis. 

Numerous epidemiological studies have shown that hyperuricemia is common in people with CVD or at high risk, such as those with hypertension, CAD, stroke, heart failure, metabolic syndrome, and peripheral vascular disease [9]. However, the frequent occurrence of comorbid illnesses among persons with elevated SUA levels presents a substantial hurdle in interpreting these results. This overlap makes distinguishing hyperuricemia's independent impact on CV outcomes difficult from the influence of other risk variables [10]. 

Some studies suggest a strong link with CV events, while others do not clearly show a cause-and-effect relationship. This inconsistency and the lack of large, randomized controlled trials specifically targeting asymptomatic patients fuel ongoing debate about whether pharmacologic treatment is beneficial, or even necessary, in the absence of clinical manifestations like gout or UA nephropathy.

In a study conducted by Zhang et al. [11] involving 607 premenopausal women, it was found that individuals with hyperuricemia exhibited a significantly higher prevalence of trivascular coronary artery disease, suggesting a potential link between elevated SUA levels and the extent of coronary involvement. Similarly, research by Dai et al. [12] demonstrated that among patients younger than 45 years of age, a SUA level exceeding 8 mg/dL was a predictive marker for the presence of trivascular disease, further supporting the association between hyperuricemia and the severity of coronary artery pathology in younger populations.

Tasic et al. [13] also observed a positive association between elevated UA levels and coronary lesions affecting two or three major vessels. However, this association lost statistical significance in multivariate analysis, indicating that while UA may be linked to coronary disease severity, its predictive value may be confounded by other coexisting CV risk factors. These findings underscore the need for further investigation into the independent role of UA in the progression and severity of multivessel coronary artery disease. From a clinical perspective, patients who have consistently elevated SUA may benefit from more regular testing and clinical follow-up. Patients with consistently high SUA levels may also require more frequent monitoring and greater clinical oversight. This could act as an effective risk assessment method, aiding in identifying those at higher CV risk who might benefit from more intense lifestyle changes. For patients with marginal CV profiles, increased SUA levels might also reinforce the choice to start preventive treatments. Emerging data suggest that hyperuricemia, especially in patients with uncontrolled gout, may play an essential role in the development and progression of cardiometabolic comorbidities [14]. Several studies have found that elevated blood uric acid (SUA) levels, which can induce hyperuricemia and gout [15], are also linked to an increased risk of CVD and all-cause mortality. Notably, the Pressioni Arteriose Monitorate E Loro Associazioni (PAMELA) study found a statistically significant increase in the risk of CV mortality for every 1 mg/dL increase in SUA, emphasizing SUA's prognostic value as a CV risk marker. Gout and CVD have similar underlying processes of systemic inflammation and oxidative stress, which accelerate atherosclerosis and contribute to poor CV outcomes. Furthermore, hyperuricemia has been associated with endothelial dysfunction and lipoprotein oxidation within atherosclerotic plaques, both of which are essential factors in the development of CVD [16].

UA has a dual biological activity, acting as an antioxidant or a pro-oxidant, depending on the microenvironmental conditions [17]. In physiological circumstances, UA can scavenge reactive oxygen species (ROS) and act as a protective antioxidant. However, under pathological or ischemic conditions, its function shifts to promote oxidative stress. During ischemia, the enzyme xanthine oxidase becomes catalytically active and uses molecular oxygen instead of nicotinamide adenine dinucleotide (NAD⁺) as an electron acceptor. This generates superoxide anions and hydrogen peroxide, both potent reactive oxygen species.

The buildup of these reactive compounds plays a crucial role in endothelial dysfunction. One important mechanism is the inactivation of nitric oxide (NO), a vital vasodilator that regulates vascular tone and endothelial integrity. Oxidants reduce NO's bioavailability and impair blood vessel vasodilation capacity. This NO depletion results in a pro-inflammatory vascular milieu characterized by leukocyte adhesion, increased permeability, and smooth muscle proliferation.

Such endothelial dysfunction not only impairs normal arterial function but also promotes atherogenesis, which contributes to the pathogenesis of CVD, such as hypertension, CAD, and heart failure. As a result, in oxidative or ischemic situations, increased UA functions as a pro-oxidant by interacting with xanthine oxidase and promoting ROS, allowing CVD to begin and advance [18].

Several studies, including Mendelian randomization analyses, have found a strong link between high blood uric acid (SUA) levels and an increased risk of CV and sudden cardiac mortality. These findings imply that elevated SUA may not only be a secondary marker of poor CV health but also play a direct, causal role in the pathogenesis of fatal CV events [19].

A Swedish cohort study (AMORIS) of 339,604 adults aged 30-60 who had no CV illness at baseline discovered that elevated UA levels were associated with an increased risk of developing AF over a 25.9-year follow-up period. AF risk increased dose-dependently compared to the lowest UA quartile, with hazard ratios of 1.09, 1.19, and 1.45 for the second, third, and fourth quartiles, respectively. This connection is maintained regardless of other illnesses such as hypertension or diabetes [20], and it was confirmed in patients who had multiple UA assessments.

Most previous research on UA and AF was cross-sectional. However, this investigation discovered that increased UA levels were associated with AF risk regardless of whether people developed CVD during follow-up. This shows that UA may have a direct role in AF development, rather than simply serving as a marker of cardiometabolic stress. The observed dose-response relationship lends evidence to this potential independent link. The data support SUA as a marker for CVD and a measure of CV risk factor load.

Multiple studies have shown a link between high blood uric acid (SUA) levels and the development of AF. A prospective cohort study of 123,238 Chinese people enrolled between 2006 and 2014 found that both high baseline SUA levels and growing SUA levels over time were associated with an increased incidence of AF [10].

Elevated UA levels in midlife are associated with an increased chance of developing AF later in life. This risk appears to be indirect (by CVD) and may be direct (via other mechanisms). A Japanese study with a 13.9-year follow-up found that elevated UA is an independent risk factor for AF, even after accounting for other heart risk factors [9]. Because elevated UA is frequently associated with being overweight, lifestyle adjustments such as diet and exercise are crucial. However, further research is needed to determine whether reducing UA levels with medication can help prevent AF in the general population.

UA has been demonstrated to promote inflammation by activating proinflammatory cytokines, and inflammation is thought to play an essential part in the underlying processes of AF. Numerous studies support the notion that inflammation contributes to specific kinds of AF, with inflammatory indicators such as C-reactive protein associated with an increased risk of AF in population-based studies [21].

In addition to its pro-inflammatory properties, UA is linked to the stimulation of oxidative stress, a major contributor to CVD and AF development. Elevated UA levels have been demonstrated to impede the generation of nitric oxide (NO) in endothelial cells, limiting vasodilation and causing endothelial dysfunction, an early stage in atherogenic and arrhythmogenic processes [22]. Reduced NO availability inhibits vascular relaxation while increasing the atrial substrate's sensitivity to remodeling and arrhythmia. Furthermore, UA promotes VSMC proliferation by activating the vascular renin-angiotensin system (RAS), which is involved in blood pressure regulation and vascular remodeling [22,23]. This UA-induced activation of RAS causes arterial stiffening [24], which may increase left atrial pressure and size, which are known risk factors for AF.

Increased arterial stiffness has been linked to defective ventricular-atrial coupling and atrial structural alterations, which increase the risk of atrial electrical instability and arrhythmogenesis. As a result, the contribution of UA to oxidative stress, endothelial dysfunction, RAS activation, and vascular remodeling creates a multifactorial process that may relate hyperuricemia to the start and progression of AF. Historically, hyperuricemia's impact on CV illness was disregarded, but new data support its position as a contributing CV risk factor. Elevated SUA levels can cause inflammatory processes and oxidative stress, resulting in endothelial dysfunction, a vital early stage in the development of atherosclerosis [24]. Increased SUA levels most likely contributed to the progression of this patient's CAD. Although current international guidelines recommend not treating asymptomatic hyperuricemia unless specific risk factors are present, this case suggests that addressing SUA levels may have been beneficial, particularly given the patient's comorbid conditions, which included hypertension, diabetes, and unstable angina.

Studies indicate that greater UA levels around middle age are connected with an increased chance of developing AF later in life. This increased risk could be explained not only by the presence of CVD and its associated risk factors but also by UA's potential direct impact on AF development via other routes. More research is needed to understand these pathways better and evaluate, via randomized controlled trials, if reducing UA levels can help prevent AF in the general population.

## Conclusions

This case suggests the complex link between asymptomatic hyperuricemia and the development of CVD, especially in patients with pre-existing risk factors, including hypertension and type 2 diabetes mellitus. Although hyperuricemia has traditionally been connected with gout, its importance as an independent risk factor for CVD is becoming increasingly recognized. The absence of overt gout symptoms resulted in consistently underestimating the increased blood uric acid (SUA) levels. Finally, the patient acquired significant multivessel CAD, necessitating CABG, raising the potential that earlier detection and care of hyperuricemia could have helped to prevent or delay ischemic heart disease.

Future studies are needed to better understand UA's role in CV pathophysiology and determine whether targeted pharmacologic intervention in asymptomatic individuals can improve long-term outcomes. Clinicians should incorporate SUA measurements into traditional CV risk assessment procedures, especially for patients with metabolic syndrome, chronic renal disease, or poorly controlled hypertension and diabetes. Finally, early detection, patient education, and comprehensive hyperuricemia therapy could be essential but underutilized methods in the prevention and treatment of ischemic heart disease. Healthcare providers can minimize CV risk and improve survival in vulnerable populations by monitoring and treating asymptomatic hyperuricemia.
